# Blood Flow Restriction Training in Seniors: An Alternative to High-Load Resistance Training

**DOI:** 10.3390/jcm15208079

**Published:** 2026-10-17

**Authors:** Krzysztof Kasicki, Grzegorz Błażejewski

**Affiliations:** 1 Department of Sport Theory and Motor Skills, Institute of Sports Sciences, University of Physical Culture, 31-571 Kraków, Poland; 2 Faculty of Health Sciences, Andrzej Frycz Modrzewski Krakow University, 30-705 Kraków, Poland

**Keywords:** blood flow restriction, older adults, resistance training, sarcopenia, muscle strength, muscle hypertrophy

## Abstract

**Background:** High-load resistance training is the primary intervention to counteract sarcopenia; however, it may be contraindicated or poorly tolerated in some older adults. Low-load resistance training with blood flow restriction (LL-BFR) has been proposed as an option for these individuals. This review compared LL-BFR with high-load resistance training in older adults. **Methods:** The review followed the PRISMA 2020 and SWiM guidelines. PubMed, Web of Science, Scopus, and EBSCOhost were searched for controlled trials (sample mean age ≥ 60 years) with an intervention lasting ≥4 weeks and a parallel high-load arm (≥60% 1RM). Risk of bias was assessed with RoB 2, study-level effects were expressed as Hedges’ g without statistical pooling, and the certainty of evidence was rated using GRADE. **Results:** Nine randomized trials (*n* = 198) were included. For dynamic maximal strength, the point estimates available from four trials were in the direction of high-load training (g 0.04 to 1.14), but confidence intervals were wide, and only one excluded no difference; isometric and isokinetic estimates were inconsistent in direction (g −0.77 to 0.79). For muscle mass and cross-sectional area, study-level estimates ranged from −0.15 to 0.42, and all confidence intervals included no difference. The BFR dose was incompletely reported in four trials. The certainty of evidence was low for muscle mass and very low for strength and functional outcomes; these ratings are upper bounds, because publication bias could not be assessed. **Conclusions:** No clear difference between LL-BFR and high-load training was observed for muscle mass, whereas dynamic strength outcomes tended to favor high-load training; these data do not establish equivalence. LL-BFR may be considered cautiously when high loads are unsuitable, although no included trial enrolled individuals in whom high-load training was contraindicated. Registration: PROSPERO CRD420261484121.

## 1. Introduction

The proportion of older adults in the populations of developed countries is increasing steadily, and with it the importance of interventions that preserve physical function. Age-related loss of muscle mass and strength is captured by the diagnosis of sarcopenia. The European Working Group on Sarcopenia in Older People defines sarcopenia primarily by low muscle strength, confirmed by low muscle quantity or quality [1]. The global prevalence of sarcopenia in individuals aged 60 years and older is estimated at 10–27% [2]. The clinical relevance of sarcopenia extends beyond the loss of muscle tissue, because low muscle strength is a key determinant of functional independence in older adults.

Resistance training remains the intervention with the best-documented effectiveness in counteracting this process. The World Health Organization guidelines on physical activity recommend that older adults perform strengthening exercises at least twice a week [3]. The National Strength and Conditioning Association position stand on resistance training for older adults indicates that strength gains are greatest with loads approaching 70–85% of one repetition maximum [4], and a dose–response meta-analysis in this population indicated optimal loads of 70–79% of one repetition maximum, performed twice a week in two to three sets of seven to nine repetitions [5].

However, the feasibility of high-load training depends on individual functional capacity, comorbidity, and clinical status rather than on chronological age. Many older adults, including frail individuals, tolerate progressive high-load training when it is appropriately supervised [4]. In a subgroup of patients, however, these recommendations are difficult to implement. External loads of 70–85% of one repetition maximum generate high shear and compressive forces within the joints, which limits their application in individuals with osteoarthritis experiencing high pain levels or after complex orthopedic procedures. These limitations are compounded by behavioral barriers, including the fear of injury during high-load exercises and the resulting lower adherence to the training program [4]. Consequently, there is a need for methods that may induce adaptations similar to those elicited by high-load training in individuals for whom such loads are contraindicated, poorly tolerated, or not yet attainable.

Blood flow restriction training has attracted particular interest in recent years; it involves performing exercise with the simultaneous application of external compression restricting arterial blood inflow and venous outflow from the working muscle. Experimental studies have shown that low-load resistance exercise combined with moderate vascular occlusion induces greater increases in strength and muscle cross-sectional area than identical exercise performed without restriction [6]. Mechanisms proposed in the literature include, for example, exacerbated metabolic stress resulting from the accumulation of metabolites under conditions of restricted washout and the earlier recruitment of motor units in response to accumulating fatigue [7]. Low-load exercise performed under blood flow restriction (BFR) conditions has also been shown to increase S6K1 phosphorylation and the rate of muscle protein synthesis, a response potentially similar to that elicited by high-load exercise [8].

The magnitude of the stimulus resulting from BFR is not, however, a uniform quantity. It depends on several variables, primarily on whether the pressure has been individualized relative to the arterial occlusion pressure, measured in a given participant [9]. Cuff dimensions are also relevant: a wider cuff occludes blood flow in the vessel lumen at a lower pressure than a narrow cuff, hence the same pressure value may correspond to different levels of restriction [10]. General safety data from a national survey of training facilities, which was not restricted to older users, indicate that adverse events are mostly mild and transient and that thrombotic incidents are rare [11]. Evidence specific to older adults is more limited. A meta-analysis in older adults found that acute heart rate and blood pressure responses to low-load BFR exercise did not differ from those observed during high-load exercise [12]; however, systematically collected adverse event data in older adults with chronic diseases remain scarce. In musculoskeletal rehabilitation, where samples were not restricted to older adults, a meta-analysis showed greater strength gains than after low-load training without blood flow restriction, with better tolerance than high-load training [13].

Six previous systematic reviews with meta-analyses compared low-load BFR training with high-load training; their characteristics are summarized in Table 1. Reviews including adults of all ages yielded divergent findings for strength: two favored high-load training [14,15], whereas one found no significant difference [16]; changes in muscle mass did not differ significantly between modalities [14,15]. Reviews restricted to older adults included only healthy participants, and trials with a high-load training arm formed a subset of studies using various comparators, such as low-load training without blood flow restriction, walking, or no training [17,18,19]. These syntheses therefore show that BFR induces training adaptations in older adults, but they do not establish whether it represents a comparable alternative to high-load training in older adults, including those with clinical conditions in whom high loads may be contraindicated or poorly tolerated.

The present review differs from these syntheses in several respects (Table 1). Only trials with a parallel high-load training comparator were eligible, and the population was not restricted to healthy individuals; five of the nine included trials enrolled clinical populations or individuals with functional limitations, whereas previous reviews of older adults were restricted to healthy participants [17,18,19]. Strength outcomes were synthesized separately by test modality; this issue had previously been examined only in a review including adults of all ages [14]. In addition, the BFR dose was characterized, and the completeness of its reporting was assessed for each trial. Trials published in 2024–2025, after the search dates of most previous reviews, were included, and the certainty of evidence was assessed separately for each outcome.

The answer to this question is further complicated by the method of outcome measurement. Muscle strength is not a quantity measured independently of the test used, and a result obtained in a test replicating a training exercise of one of the compared arms may reflect the specificity of motor task learning [20]. The combined consideration of the comparison group selection, the method of describing the restriction dose, and the specificity of the test relative to the training modality delineates the scope of this review.

The aim of this systematic review was to summarize the evidence from controlled trials in which low-load training with blood flow restriction was compared with high-load resistance training in older adults (sample mean age ≥ 60 years). No equivalence or non-inferiority margin was prespecified; the review therefore addresses comparative effectiveness rather than formal equivalence.

The specific objectives included:

- Compiling study-level effect sizes for the primary outcomes: muscle strength and muscle mass or cross-sectional area.
- Exploring, in a hypothesis-generating manner, whether the observed difference between modalities varies according to the specificity of the test relative to the training modality.
- Describing the blood flow restriction dose and the completeness of its reporting in the included studies.

## 2. Materials and Methods

The systematic review was conducted in accordance with the Preferred Reporting Items for Systematic Reviews and Meta-Analyses (PRISMA) 2020 guidelines [21]; the completed PRISMA 2020 checklist is provided in Table S1 (Supplementary Materials). Because a meta-analysis was not considered appropriate (Section 2.6), the synthesis was reported according to the Synthesis Without Meta-analysis (SWiM) guideline [22]. The review was registered in PROSPERO (CRD420261484121). The comparator was restricted to high-load resistance training before data extraction and effect size calculation. The planned subgroup analyses according to exercise modality and sex were not performed, because no included trial used aerobic exercise with BFR and no trial reported sex-stratified data. There were no other deviations from the registered protocol.

During the preparation of this manuscript, the authors used the LLM model [Gemini 3.1 Pro] for translation and grammatical correction. The authors have reviewed and edited the output and take full responsibility for the content of this publication.

### 2.1. Search Strategy

Before the main search, one author (K.K.) conducted a preliminary search in PubMed to identify the terminology used in the literature on blood flow restriction training in older adults and to establish the keywords. The search strategy was constructed using a three-block structure, combining the population block, intervention block, and exercise-context block with the conjunction operator. The outcome block was not included in the main search because its addition reduced sensitivity below an acceptable level.

The population block was kept broad because age descriptors are indexed differently across databases, and a substantial proportion of studies on blood flow restriction in older adults do not include an age-related term in the title or abstract. Therefore, verification of the age criterion was transferred to the screening stage.

The search was conducted on 30 June 2026 in four databases: PubMed, Web of Science Core Collection, Scopus, and EBSCOhost. Within the EBSCOhost platform, six constituent databases were searched simultaneously (MEDLINE Ultimate, MEDLINE, Academic Search Ultimate, Health Source: Nursing/Academic Edition, APA PsycArticles, and OpenDissertations), thereby ensuring coverage of gray literature in the form of doctoral and master’s theses. No language or date restrictions were applied. Clinical trial registries (e.g., ClinicalTrials.gov and the WHO International Clinical Trials Registry Platform) were not searched because the review question concerned completed trials reporting training outcomes, which are indexed in bibliographic databases; this decision may have reduced the completeness of the search and limited the assessment of non-reporting bias (Section 3.7). In addition, the reference lists of included studies and identified systematic reviews were screened. The search was last updated on 31 July 2026, and no new publications meeting the eligibility criteria were identified.

The full search strategies for all four sources, presented in a reproducible format, are provided in Appendix A.

### 2.2. Study Selection and Eligibility Criteria

**Figure 1:**
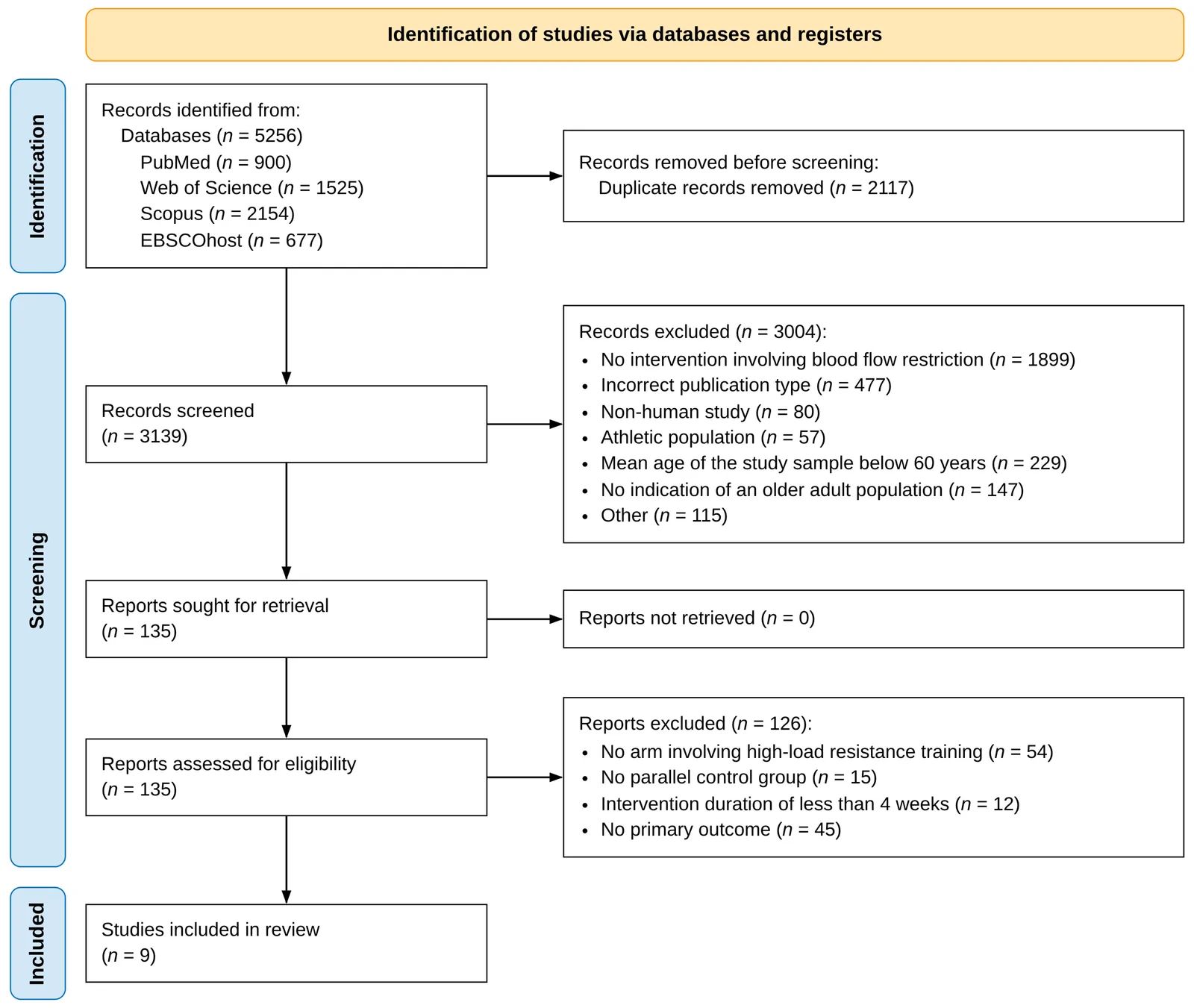
PRISMA flow chart.

Records were exported from four sources and deduplicated using a multi-pass method based on the sequential algorithm by Bramer et al. [23]. Automatic exclusion of records by publication type, language, or date was not applied. Title and abstract screening was performed by a single author (K.K.) and was not duplicated. At the full-text stage, two authors (K.K. and G.B.) independently assessed the full texts against the eligibility criteria. In cases where an unequivocal resolution was not possible, the decision was made in consultation with a third assessor (T.A.), who did not participate in the preparation of this paper. Inter-rater agreement at the full-text stage was expressed using Cohen’s kappa coefficient (κ = 0.68). At the title and abstract screening stage, the rule of inclusion in case of doubt applied, and ambiguous records, including those lacking an abstract, were directed to full-text assessment. Each record excluded at this stage was assigned one exclusion code from a hierarchical set of codes defined before screening; these codes correspond to the categories reported in Section 3.1 and Figure 1. Age-related codes were applied only when the title or abstract reported the age of the sample or explicitly described a non-older population; otherwise, the record was forwarded to full-text assessment. Each report excluded at the full-text stage was assigned one reason for exclusion, in the following order: incorrect study design, incorrect intervention, lack of an eligible comparator, insufficient intervention duration, and lack of an eligible outcome.

The publication containing the primary outcome and the largest sample was designated as the main report, while the remaining ones were treated solely as a source of supplementary data; one item per cohort was counted towards the number of included studies. Cohort overlap was identified based on the concordance of the bioethics committee approval number and baseline characteristics. Eligibility criteria, defined a priori within the PICOS framework, are summarized in Table 2.

The age criterion was applied to the sample mean because individual-level age data were rarely reported and a participant-level criterion could not be verified consistently. Consequently, individuals younger than 60 years could have been enrolled in four trials whose eligibility criteria permitted younger participants (Section 3.2). Randomized and non-randomized controlled trials were eligible a priori, and the ROBINS-I tool [24] was planned for non-randomized studies; however, no non-randomized trial met the remaining eligibility criteria.

No language or time restrictions were applied. However, interventions other than blood flow restriction training, e.g., ischemic pre- and postconditioning, were excluded. Populations undergoing postoperative and perioperative rehabilitation, as well as those treated in acute care settings, were excluded. Studies assessing solely the acute effect of a single session did not meet the inclusion criteria and are not included in the flow diagram or in the synthesis; this literature was used in the Discussion as material regarding the potential mechanisms of BFR effects.

### 2.3. Data Extraction

Data extraction was performed by one author (K.K.), and the extracted data were verified against the source publications by a second author (G.B.). Data were extracted from the full texts using a standardized form in an Excel spreadsheet (Microsoft Corporation, Redmond, WA, USA), which was piloted on two studies before the main extraction, and were organized into four blocks:

- Study characteristics: country, setting, study design, method of allocation to study arms, sample size in each arm at randomization and in the analysis;
- Participant and intervention characteristics: population, including diagnostic criteria, age, duration and frequency of the intervention, exercise load in the blood flow restriction arm and the comparator arm, and type of exercise;
- Blood flow restriction dose parameters: cuff width and length, pressure value in mmHg, percentage of arterial occlusion pressure, method used to determine it, and whether pressure was maintained or released during rest intervals between sets;
- Outcomes: test and unit used, mean values and standard deviations before and after the intervention for each arm, results of tests of statistical significance for within- and between-group differences, effect sizes reported by the authors, as well as adverse events, and number and reasons for dropouts.

Completeness of reporting of the blood flow restriction dose was considered adequate when cuff width, pressure value, and the method used to determine the pressure were reported simultaneously; incomplete reporting was not an exclusion criterion. The time point for the primary analysis was the measurement performed immediately after completion of the intervention, whereas delayed measurements were recorded separately and were not included in the synthesis.

Standard deviations were derived from the standard error or from the limits of the 95% confidence interval in accordance with Chapter 6 of the Cochrane Handbook [25].

### 2.4. Risk of Bias Assessment

Risk of bias was assessed using the Cochrane Risk of Bias 2 (RoB 2) tool [26] across five domains: the randomization process, deviations from intended interventions, missing outcome data, measurement of the outcome, and selective reporting of the result. The assessment was applied to the primary outcome, which is muscle strength, and to the effect of assignment to the intervention. The assessments were independently conducted by two authors (K.K. and G.B.), and discrepancies were resolved through discussion, with the involvement of a third assessor (T.A.), who was not involved in the preparation of this paper, when consensus could not be reached. Because all included studies randomly allocated participants to the compared arms, RoB 2 was applied, and the ROBINS-I tool [24] was not required. Blinding of participants and training personnel to the compression cuff is not feasible in BFR trials. In the domain of deviations from intended interventions, the lack of blinding was therefore not equated with a high risk of bias; judgments were based on whether deviations arose because of the trial context and whether an appropriate analysis was used. When the information required to answer a signaling question was not reported, the domain was judged as raising some concerns.

### 2.5. Effect Measures

The effect measure for the outcomes was the standardized mean difference in change from baseline, expressed as Hedges’ g along with a 95% confidence interval [27]. A convention was adopted in which positive values indicate an advantage of high-load training, and negative values indicate an advantage of blood flow restriction training. The standard deviation of the change was calculated from the standard deviations before the intervention and after its completion, assuming a within-participant correlation of r = 0.7, in accordance with the Cochrane Handbook recommendations [25]; if the authors reported the change along with a 95% confidence interval, the standard deviation of the change was reconstructed from that interval. The value of r = 0.7 was adopted as a central assumption; within-participant correlations back-calculated from the trial arms that reported pre-intervention, post-intervention, and change standard deviations ranged from 0.32 to 0.92 (Section 3.5.1), and sensitivity analyses were therefore performed at r = 0.5 and r = 0.9 (Section 2.7). Hedges’ correction for small samples was applied to all estimates. In a study comprising two blood flow restriction arms differing in the magnitude of occlusion pressure, these arms were combined into a single intervention arm to avoid multiple uses of the same comparison group. All calculations were performed in the R environment version 4.3.3 (R Foundation for Statistical Computing, Vienna, Austria) [28].

### 2.6. Synthesis Methods

A meta-analysis was considered but was not performed because the included trials did not provide a sufficient basis for a reliable pooled effect estimate. The number of independent comparisons was small: a standardized effect size could be calculated for four of the six trials assessing dynamic maximal strength and for three of the four trials assessing muscle mass or muscle cross-sectional area. With so few studies available, the estimation of between-study variance would have been subject to considerable uncertainty. Moreover, the ability to assess heterogeneity reliably would have remained limited. These difficulties would have been further compounded by the dependence among individual estimates described below [25]. Therefore, meta-analysis was not considered methodologically impossible but rather an approach unlikely to yield a reliable and unambiguously interpretable pooled estimate. An additional concern was that the missing variance data might not have been random with respect to the observed effects. In the two studies for which effect sizes could not be calculated, the reported between-group differences were close to zero or favored BFR. Restricting quantitative synthesis to studies providing sufficient data to calculate effect sizes could therefore have resulted in a biased pooled estimate. Furthermore, several trials contributed multiple dependent estimates, including results from three dynamic strength tests in one sample and measurements of both limbs in another. Outcome definitions differed within categories, including quadriceps cross-sectional area measured by magnetic resonance imaging versus appendicular skeletal muscle mass index estimated by bioelectrical impedance, as well as directly measured versus estimated one-repetition maximum. Differences in BFR pressure, exercise type, and population characteristics further increased clinical heterogeneity.

The synthesis was therefore conducted according to the SWiM guideline [22]. Studies were grouped by outcome, and strength outcomes were additionally grouped by test modality. Study-level effect sizes with 95% confidence intervals were tabulated and described in terms of direction, magnitude, and precision. Vote counting based on statistical significance was not used for inference. Ranges of effect sizes reported in the Results refer to study-level estimates and do not represent pooled effects. Comparisons between test modalities and the within-trial comparison of two BFR pressures were regarded as descriptive and hypothesis-generating.

### 2.7. Sensitivity Analyses and Publication Bias

The calculations were repeated with within-participant correlations of r = 0.5 and r = 0.9. Another sensitivity analysis consisted of separating the combined intervention arms in a multi-arm study and calculating the effect size separately for the higher and lower occlusion pressure arms. Both analyses evaluated whether the direction of the estimates remained unchanged. Graphical and statistical methods used to detect small-study effects require at least ten studies within a single comparison [25]. Because this condition was not met, neither a funnel plot nor Egger’s test was used, and the risk of bias due to selective reporting was assessed qualitatively using the following criteria: lack of prospective study registration, presence of companion publications reporting selected endpoints of the same cohort in separate papers, and incompleteness of reporting within the included studies.

### 2.8. Certainty Assessment

The certainty of the evidence was assessed using the Grading of Recommendations Assessment, Development and Evaluation (GRADE) approach [29]. Because no meta-analysis was conducted, ratings were based on the body of study-level estimates for each outcome, in line with GRADE guidance for rating certainty in the absence of a single effect estimate [30]. As all included studies were randomized, the rating started at high certainty and was downgraded by one level for serious and by two levels for very serious concerns, according to the following operational criteria. Risk of bias: most of the evidence came from studies judged as raising some concerns or at high risk of bias. Inconsistency: point estimates differed in direction, and at least one confidence interval excluded no difference in the opposite direction; magnitudes ranged from trivial to large without a plausible explanation, or instruments were too diverse to judge consistency; structural heterogeneity in outcome definitions was considered under this domain. Indirectness: the outcome measure or population departed from the review question. Imprecision: Because no pooled confidence interval was available, a pragmatic criterion for assessing imprecision was applied, which did not constitute a formal GRADE rule. Imprecision was considered serious when the total sample size was below approximately 400 participants, the threshold below which GRADE guidance recommends considering downgrading the certainty of evidence for imprecision for continuous outcomes [31] and when most confidence intervals reported in individual studies included both the null effect and effects of |g| ≥ 0.5, corresponding to at least moderate effect sizes and leading to different interpretations. Meeting these criteria resulted in downgrading the certainty of evidence by one level. For evidence based on a single study, certainty was downgraded by two levels.

The certainty of evidence was not downgraded for potential publication bias because each synthesis included fewer than ten studies, precluding its reliable assessment using standard formal methods. However, the absence of downgrading in this domain should not be interpreted as evidence of the absence of publication bias. Consequently, the certainty ratings presented in this review should be regarded as potential upper bounds of the true certainty, which may be lower.

The reason for each downgrade is given in the footnotes to the GRADE evidence table (Section 3.6). Assessments were performed independently by two authors (K.K. and G.B.), and discrepancies were resolved by a third assessor (T.A.), who served as an independent reviewer and was not involved in the preparation of this manuscript.

## 3. Results

### 3.1. Study Selection

The four databases yielded 5256 records: PubMed (*n* = 900), Web of Science Core Collection (*n* = 1525), Scopus (*n* = 2154), and EBSCOhost (*n* = 677). After deduplication, 2117 records were removed.

During title and abstract screening, 3004 records were excluded; each record was assigned one code according to the hierarchy defined before screening (Section 2.2), so the categories below sum to the number of excluded records (Figure 1). The largest category was the absence of an intervention involving blood flow restriction (*n* = 1899). Other categories included an incorrect publication type (*n* = 477), a mean age of the study sample below 60 years (*n* = 229), no indication of an older adult population (*n* = 147), studies not conducted in humans (*n* = 80), and studies involving athletic populations (*n* = 57). The following criteria—acute effects (*n* = 34), ischemic pre- and postconditioning (*n* = 43), and terminology referring to clinical applications unrelated to exercise training (*n* = 38)—were collectively classified as “Other (*n* = 115)” in the PRISMA flow diagram.

Of the 135 reports assessed for eligibility in full text, 126 were excluded because of the absence of a parallel arm using high-load resistance training (*n* = 54), absence of a parallel control group (*n* = 15), intervention duration of less than four weeks (*n* = 12), or absence of the primary outcome (*n* = 45). Ultimately, nine studies were included in the review.

It was additionally noted that the studies by Vechin et al. [32] and Libardi et al. [33] most likely share the same control group: both studies report a group of *n* = 7, with a mean age of 66.0 ± 5.0 and 65.0 ± 4.0 years, a body mass of 69.3 ± 16.1 and 69.2 ± 15.1 kg, respectively, and an identical height of 1.61 ± 0.1 m. Because the primary analysis does not use the control groups, this observation does not affect the comparisons presented; however, it was noted because of its relevance to the assessment of the independence of the evidence. The intervention arms of the two trials were distinct (resistance training alone [32] versus concurrent training [33]); nevertheless, both trials originated from the same research group, their results are not considered fully independent, and the overlap is flagged in Table 3. The study selection process is presented in Figure 1.

**Table 3:** Characteristics of the included studies.

Study	Country	Arm Size BFR/HL/C	Population	Age BFR/HL (Years)	Duration (Weeks)	Sessions (Per Week)	BFR Load (% 1RM)	Comparator Load (% 1RM)	Outcomes Measured
Vechin et al. [32] ^a^	Brazil	8/8/7	Healthy individuals	65.0 ± 2.0/62.0 ± 3.0	12	2	20 → 30	70 → 80	Leg press 1RM; quadriceps cross-sectional area (MRI)
Libardi et al. [33] ^a^	Brazil	10/8/7	Healthy individuals	64.0 ± 4.0/65.0 ± 3.7	12	2	20 → 30	70 → 80	Leg press 1RM; quadriceps cross-sectional area (MRI)
Silva et al. [34]	Brazil	5/5/5	Women with osteoporosis	62.6 ± 4.3/61.8 ± 6.0	12	2	30	80	Knee extension 1RM
Bryk et al. [35]	Brazil	17/17/–	Women with knee osteoarthritis	62.3 ± 7.0/60.4 ± 6.7	6	3	30	70	Quadriceps MVC; Lequesne; TUG; NPRS
De Sá et al. [36]	Brazil	13/11/–	Women with osteopenia	65.9 ± 10.4/66.0 ± 5.8	8	2	30	70	Leg press 1RM
Cook et al. [37]	USA	12/12/12	Individuals at risk of mobility limitation	76.5 (72.3–80.7)/76.7 (71.3–82.0)	12	2	30 (50 for leg press)	70	1RM (3 exercises); MVIC; quadriceps cross-sectional area (MRI); SPPB
Kim et al. [38]	USA	9/10/–	Healthy individuals	63.0 ± 0.8/62.5 ± 1.1	4	3	20 (% MVC)	75 (% MVC)	Handgrip MVC; forearm circumference; forearm blood flow
Zhang et al. [39]	China	10/11/–	Individuals with sarcopenia	71.3 ± 3.2/73.2 ± 4.2	12	3	20 → 30	60 → 65 → 70	Knee extension 1RM; ASMI; handgrip strength; SPPB
Letieri et al. [40]	Brazil/Portugal	11 + 11/10/12	Recreationally active women	68.8 ± 5.1 (entire sample)	16	3	20 → 30	70 → 80	Isokinetic torque of knee extensors and flexors

BFR—blood flow restriction arm; HL—high-load resistance training arm; C—control group without training; 1RM—one-repetition maximum; MVC—maximal voluntary contraction; MVIC—maximal voluntary isometric contraction; MRI—magnetic resonance imaging; ASMI—appendicular skeletal muscle mass index; SPPB—Short Physical Performance Battery; TUG—Timed Up and Go; NPRS—11-point numerical pain rating scale; age is reported as mean ± standard deviation. A dash indicates that the respective arm was not included in the study design. ^a^ Trials from the same research group that most likely shared a control group (Section 3.1); control groups were not used in the comparisons of this review, and the intervention arms were distinct. Companion reports of included cohorts [41,42] were used only as sources of Supplementary Data.

### 3.2. Characteristics of the Included Studies

The studies were published between 2015 and 2025: five originated from Brazilian research centers [32,33,34,35,36], two from the United States [37,38], one from China [39], and one from a Brazilian–Portuguese collaboration [40]. All nine studies were randomized; in two cases, randomization was preceded by ranking participants according to baseline quadriceps muscle cross-sectional area or strength [32,33].

The total number of participants was 198. The median sample size per arm was 10 participants, with a range of 5 to 22. The mean age of the study samples ranged from 60.4 to 76.7 years. In four trials, the eligibility criteria permitted the inclusion of individuals younger than 60 years: age ranges of 59–71 years [32], >50 years [34], and 50–70 years [36], and no lower age limit [35].

The populations were clinically heterogeneous. For the purpose of synthesis, trials were classified into two groups. Four trials enrolled healthy older adults (healthy aging): three included healthy individuals [32,33,38], and one included recreationally active women [40]. Five trials enrolled clinical or functionally limited populations: knee osteoarthritis [35], osteoporosis [34], osteopenia [36], sarcopenia [39], and individuals at risk of mobility limitation due to muscle weakness [37].

The intervention duration ranged from 4 to 16 weeks, with a median of 12 weeks, and intervention frequency ranged from two to three sessions per week. Loads in the blood flow restriction arms ranged from 20% to 50% of one-repetition maximum, whereas loads in the comparator arms ranged from 60% to 80%; in one study [39], the 65% threshold was reached only in the fifth week of the intervention. Eight studies used lower-limb resistance training; one study [38] trained the forearm muscles and assessed handgrip MVC. In one study [33], resistance training formed part of a program combined with endurance training, which was identical in both arms; the total volume of resistance training in the blood flow restriction arm was 41% lower during weeks 1–6 and 34% lower during weeks 7–12 (Table 3).

### 3.3. Characteristics of the Blood Flow Restriction Dose

Absolute cuff pressure ranged from 67 ± 8 mmHg [33] to 200 mmHg [35], while cuff width ranged from 6 cm [37] to 18 cm [32,34]. Six studies indexed pressure to individually measured arterial occlusion pressure, using values ranging from 50% to 80%. Three studies did not use this method: Bryk et al. [35] used a fixed pressure of 200 mmHg for all participants, Kim et al. [38] used 130% of systolic blood pressure, and Cook et al. [37] used approximately 1.5 times systolic blood pressure, resulting in a mean pressure of 184 ± 25 mmHg.

Reporting of the blood flow restriction dose was incomplete in four of the nine studies. Reporting was considered complete when cuff width, absolute cuff pressure, and the method used to determine the pressure were all reported. Two studies did not report cuff width [35,40], while two did not report the absolute pressure value and reported only the percentage of arterial occlusion pressure [36,39]. Maintenance of cuff pressure during rest intervals between sets was reported in seven studies, while in two studies the cuff was deflated between sets [34,36] (Table 4).

With respect to the method of prescription, pressure was individualized relative to arterial occlusion pressure in six trials (50% [32,33,39], 70% [36], and 80% [34,40]), indexed to systolic blood pressure in two trials [37,38], and fixed at an arbitrary value in one trial [35]. The dose was reported completely, and was therefore reproducible, in five trials [32,33,34,37,38]. Evidence on muscle mass and cross-sectional area was derived predominantly from protocols using 50% of arterial occlusion pressure with 14–18 cm cuffs [32,33,39]; only one estimate came from a 6 cm cuff with pressure indexed to systolic blood pressure [37].

### 3.4. Risk of Bias in Studies

**Figure 2:**
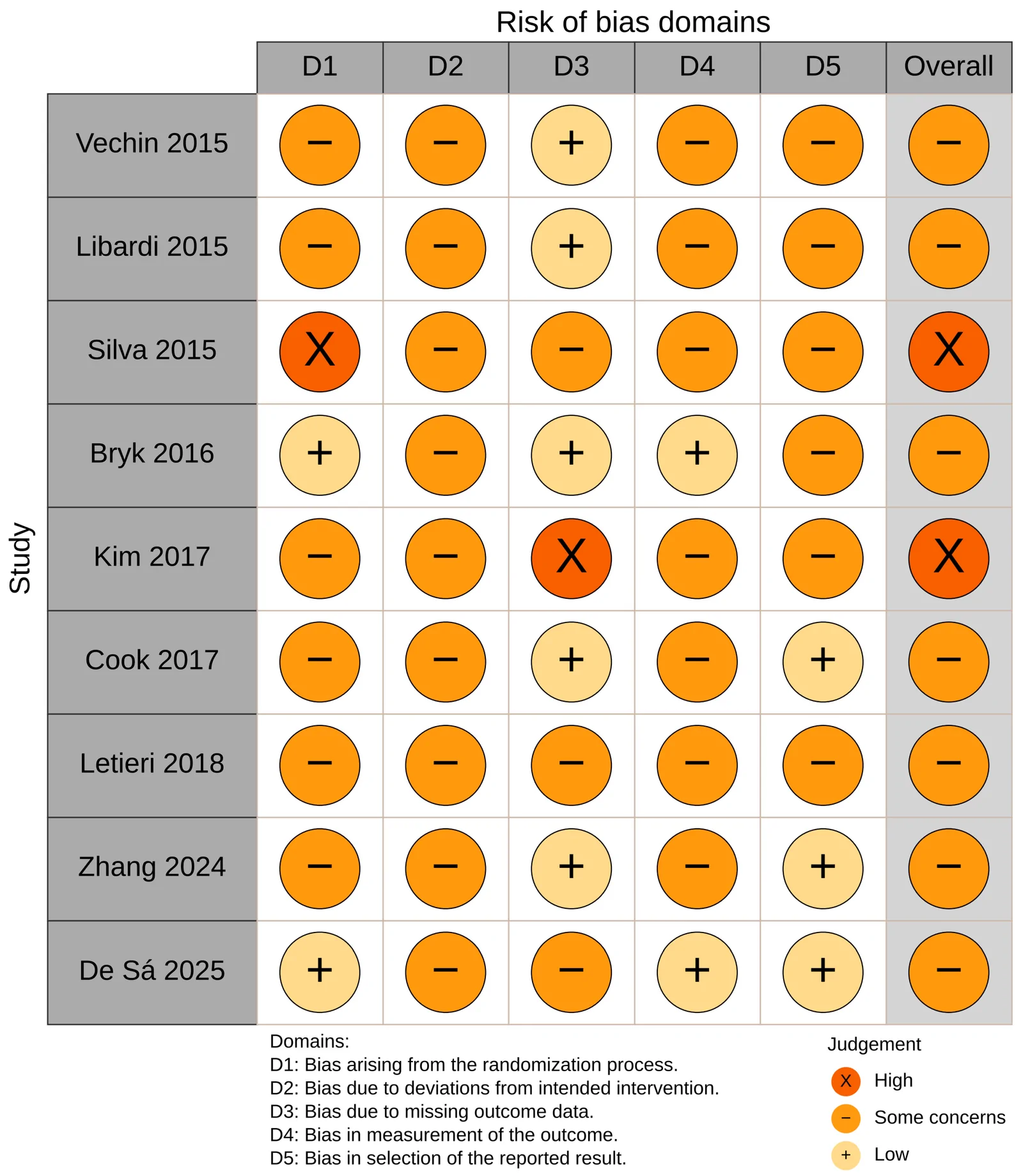
Risk of bias assessment for individual studies [32,33,34,35,36,37,38,39,40].

**Figure 3:**
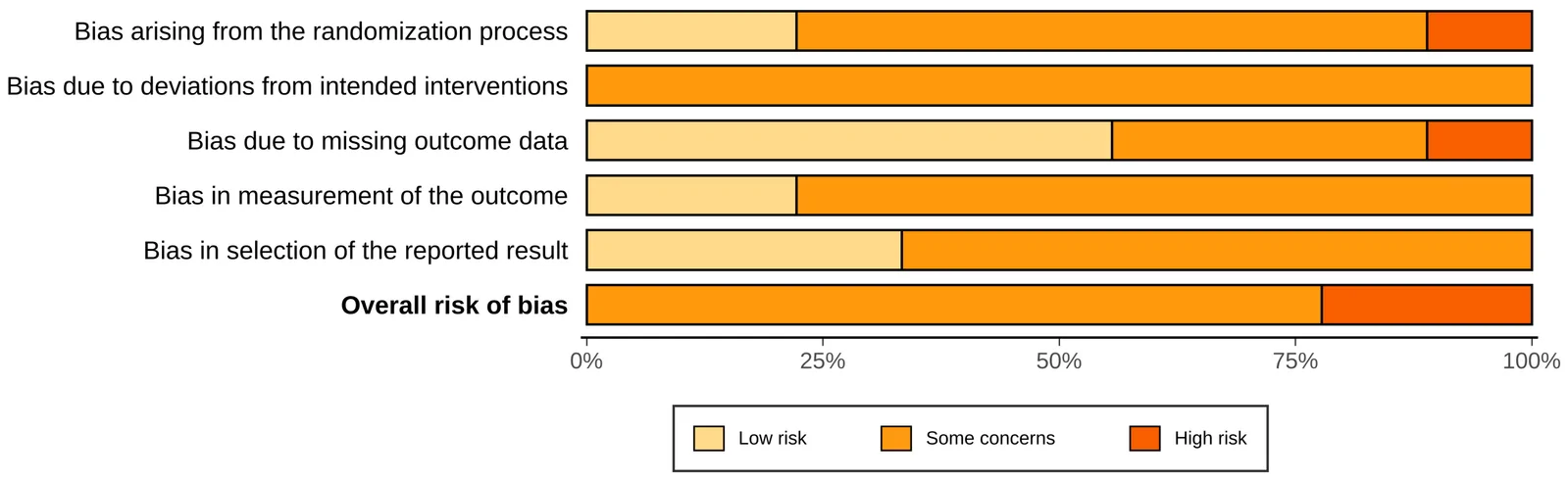
Distribution of assessments across domains.

Risk of bias was assessed using the RoB 2 tool [26] (Figure 2 and Figure 3). All nine studies included in the review randomly allocated participants to the compared arms.

None of the nine studies received an overall assessment indicating a low risk of bias. Seven studies were judged to have some concerns, while two were judged to have a high risk of bias.

In the domain of the randomization process, two studies were judged to have a low risk of bias: Bryk et al. [35] used sealed envelopes drawn by a person not involved in the study, while De Sá et al. [36] used block randomization stratified by age and muscle strength, with sealed envelopes prepared by a researcher outside the study team. Six studies were judged to have some concerns, mainly because the method used to conceal allocation was not described. One study was judged to be at high risk of bias [34], because baseline knee extension strength differed by approximately 29% between the arms, each comprising five participants.

In the domain of deviations from intended interventions, all nine studies were judged to have some concerns, because participants and training personnel were aware of the allocated intervention, and the information on deviations arising from the trial context was insufficient to exclude their influence (Section 2.4).

In the domain of missing outcome data, five studies were judged to have a low risk of bias. Three studies were judged to have some concerns. One study was judged to have a high risk of bias [38]: four of the 13 participants allocated to the blood flow restriction arm discontinued the study, corresponding to 31%, with discomfort related to the protocol itself given as the reason. In this case, the missingness was related to the intervention and was also likely related to the assessed outcome.

In the domain of outcome measurement, two studies were judged to have a low risk of bias: in Bryk et al. [35], the assessor was blinded to allocation and was not involved in delivering the intervention, while in De Sá et al. [36], the researchers collecting and analyzing the data were blinded to allocation. The remaining seven studies were judged to have some concerns because the outcome depended on effort from unblinded participants, combined with a lack of information regarding blinding of the personnel conducting the tests. The possible influence of test specificity on strength outcomes was not treated as a risk-of-bias judgment and is considered in Section 4.1.

In the domain of selective reporting, three studies were judged to have a low risk of bias [36,37,39] because of prospective registration or an explicitly stated primary objective. Six studies were judged to have some concerns because no protocol was available.

### 3.5. Intervention Effects

Quantitative pooling was not performed for the reasons given in Section 2.6; study-level effect sizes are presented without a summary estimate, in accordance with the SWiM guideline [22]. Positive values of Hedges’ g indicate a larger change after high-load resistance training.

A standardized effect size could be calculated for seven of the nine studies, comprising a total of fourteen comparisons. In two studies [33,36], results were presented using bar graphs or percentage changes together with within-group effect sizes. Attempting to reconstruct standard deviations from the reported effect sizes and subsequently using them to calculate a standardized mean difference would have resulted in methodologically unjustified inference. Therefore, for these two studies, effect sizes were presented in the form originally reported by the authors.

#### 3.5.1. Muscle Strength

All studies reported at least one measure of muscle strength (Table 5). Dynamic maximal strength was assessed in six trials contributing eight comparisons, and Hedges’ g could be calculated for six comparisons from four trials. The six point estimates, derived from only four trials, were all in the direction of high-load resistance training (g 0.04 to 1.14); however, most confidence intervals were wide, only the estimate for knee extension 1RM in Cook et al. [37], g = 1.14 (95% CI 0.27 to 2.01), excluded no difference, and the remaining estimates were compatible with no difference between modalities. Other estimates were obtained for knee extension 1RM in Silva et al. [34], g = 1.07 (−0.28 to 2.41), leg press 1RM in Cook et al. [37], g = 0.55 (−0.27 to 1.36), knee flexor 1RM in the same study, g = 0.43 (−0.38 to 1.24), leg press 1RM in Vechin et al. [32], g = 0.43 (−0.56 to 1.42), and estimated knee extension 1RM in Zhang et al. [39], g = 0.04 (−0.82 to 0.90). The authors of one study [32] independently reported an effect size of 0.92 for the comparison of high-load resistance training with blood flow restriction training, calculated from baseline-adjusted changes. In the two trials without calculable effect sizes, relative gains were similar in both arms (+35.4% vs. +38.1% [33]) or larger after BFR (+33.5% vs. +29.7% [36]).

Across five comparisons based on isometric and isokinetic tests, the results were more heterogeneous. Four estimates were in the direction of high-load resistance training: handgrip MVC in Kim et al. [38], g = 0.79 (−0.15 to 1.73), isokinetic knee extensor torque of the left and right limbs in Letieri et al. [40], g = 0.55 (−0.21 to 1.31) and g = 0.32 (−0.43 to 1.08), respectively, and extensor MVIC in Cook et al. [37], g = 0.40 (−0.41 to 1.21); all four confidence intervals included no difference. The only estimate indicating an advantage of blood flow restriction training came from Bryk et al. [35]: the increase in maximal isometric quadriceps contraction was 16.8 ± 10.3 units vs. 9.4 ± 8.3 in the high-load resistance training arm, corresponding to g = −0.77 (−1.47 to −0.07). The authors of this study reported a between-group difference in change of 7.4 (0.9 to 13.9), a confidence interval that excluded no difference, whereas the abstract and the results of the repeated-measures analysis of variance described the between-group effect as non-significant. In a design with two groups and two time points, the group-by-time interaction is equivalent to a comparison of change scores; a t-test on the reported change scores reproduced the published confidence interval (*p* ≈ 0.03). The discrepancy therefore cannot be attributed to a different analytical procedure and was regarded as an internal inconsistency of the original report; the estimate was retained as calculated.

Cook et al. [37] measured both dynamic maximal strength and maximal voluntary isometric contraction in the same sample. The effect size for the dynamic test was g = 1.14 (0.27 to 2.01), whereas that for the isometric test performed in the same participants was g = 0.40 (−0.41 to 1.21); the statistically significant difference observed in the dynamic test was not maintained in the isometric test (*p* = 0.14). Across trials, the pattern was less clear: the median effect size was 0.49 for dynamic tests and 0.40 for isometric and isokinetic tests, a descriptive comparison that was not tested formally, while the only study indicating an advantage of blood flow restriction training [35] was also the only study in which the assessor was blinded and the test did not replicate the training modality of either arm.

Within-participant correlations back-calculated from the four trial arms that reported pre-intervention, post-intervention, and change standard deviations [35,39] ranged from 0.32 to 0.92. Sensitivity analysis of the assumed within-participant correlation showed that effect sizes for strength were moderately sensitive to this assumption. For Silva et al. [34], g ranged from 0.83 at r = 0.5 to 1.77 at r = 0.9; for Kim et al. [38], from 0.61 to 1.36; and for Letieri et al. [40], from 0.25 to 0.56. The direction of the effect remained unchanged across all analyzed scenarios (Table 5).

When the two BFR arms of Letieri et al. [40] were analyzed separately in an exploratory analysis, the estimates for the higher-pressure arm were close to zero (right limb, g = −0.14; −1.00 to 0.72) or small (left limb, g = 0.30; −0.56 to 1.16), whereas those for the lower-pressure arm were larger and in favor of high-load resistance training (right limb, g = 0.85; −0.05 to 1.74; left limb, g = 0.85; −0.05 to 1.75). All confidence intervals included no difference, and the pattern was unchanged at r = 0.5 and r = 0.9.

#### 3.5.2. Muscle Mass and Cross-Sectional Area

Muscle mass or cross-sectional area was reported by four studies, three of which used magnetic resonance imaging [32,33,37] and one bioelectrical impedance analysis [39]. A fifth study [38] was limited to measurement of forearm circumference, which did not meet the criterion for the primary outcome (Table 6).

In all comparisons for which an effect size could be calculated, the confidence intervals included no difference. Increases in quadriceps cross-sectional area were similar in both arms: in Vechin et al. [32], g = 0.01 (−0.97 to 0.99), with increases of 6.6% vs. 7.9%, and in Cook et al. [37], g = −0.15 (−0.95 to 0.65), with changes of 3.23 cm^2^ vs. 2.86 cm^2^. Libardi et al. [33] reported increases of 7.6% in the blood flow restriction arm vs. 7.3% in the high-load resistance training arm, with no between-group difference. A different result was observed by Zhang et al. [39], in whom appendicular skeletal muscle mass index increased significantly only in the high-load resistance training arm (+0.30 kg·m^−2^; 95% CI 0.00 to 0.59; *p* = 0.021), compared with a non-significant increase in the blood flow restriction arm (+0.13 kg·m^−2^; −0.10 to 0.36; *p* = 0.169), corresponding to g = 0.42 (−0.45 to 1.29), with no significant between-group difference (*p* = 0.349). This was the only study to use bioelectrical impedance analysis.

Sensitivity analysis showed that the effect size for muscle cross-sectional area was insensitive to the assumed within-participant correlation; in Vechin et al. [32], g ranged from 0.01 to 0.02 across the entire range of r examined.

#### 3.5.3. Discrepancy Between Hypertrophic Adaptation and Strength Gains

In the two studies that simultaneously measured both primary outcomes using an imaging-based method, the directions of the effects diverged: in Cook et al. [37], an advantage of high-load resistance training in the dynamic strength test (g = 1.14) was accompanied by no difference in quadriceps cross-sectional area (g = −0.15), while in Vechin et al. [32], a point estimate of moderate magnitude in the direction of high-load training for strength (g = 0.43; confidence interval including no difference) was accompanied by no difference in cross-sectional area (g = 0.01). In the third imaging-based study [33], both strength and cross-sectional area changed to a similar extent in both arms.

The three calculable study-level estimates for muscle mass and cross-sectional area ranged from −0.15 to 0.42, and their confidence intervals included no difference. For muscle strength, nine of eleven study-level point estimates exceeded 0.30 in the direction of high-load resistance training, although nine of eleven confidence intervals included no difference; these counts describe the distribution of estimates and do not constitute a test of effect. Interpretation of this discrepancy is beyond the scope of the Results section and is addressed in the Discussion.

#### 3.5.4. Functional Performance

Measures of functional performance were reported by three studies. Cook et al. [37] reported a 4% increase in 400 m walking speed across all groups combined (*p* = 0.007), with no change in the overall Short Physical Performance Battery score (*p* = 0.33) or in any quality-of-life domain; however, the chair-stand component of the test improved (*p* = 0.011). Zhang et al. [39] found improvement in 6 m walking performance in both arms (BFR: +0.18; 0.07 to 0.30; HL: +0.24; 0.16 to 0.31), whereas a significant improvement in the Short Physical Performance Battery score was observed only in the high-load resistance training arm (+2.09; 1.39 to 2.79; *p* = 0.004), compared with a trend in the blood flow restriction arm (+0.60; −0.09 to 1.29; *p* = 0.059). Bryk et al. [35] reported improvements in the Timed Up and Go test and Lequesne index in both arms, with no between-group difference.

None of the studies demonstrated that increases in strength and muscle mass translated into improvements in composite measures of functional performance, and no significant between-group differences in functional performance were reported; a significant within-group improvement in the SPPB score was observed only in the high-load arm of one trial [39].

#### 3.5.5. Cardiorespiratory Fitness and Other Outcomes

Cardiorespiratory fitness was reported by one study. Libardi et al. [33] found a 10.3% increase in peak oxygen uptake in the blood flow restriction arm (*p* = 0.02) vs. 9.5% in the high-load resistance training arm (*p* = 0.04), with no difference between arms and no change in the control group (−1.1%). In both arms, resistance training was part of a combined program with endurance training of identical volume and intensity; therefore, the observed improvement cannot be attributed to blood flow restriction alone.

Metabolic and hemodynamic outcomes were also reported by one study. Zhang et al. [39] found significant reductions in systolic blood pressure in both arms (BFR: −24.30 mmHg; −41.26 to −7.34; HL: −16.09 mmHg; −30.93 to −1.25), an increase in high-density lipoprotein concentration, a reduction in triglyceride concentration, an increase in growth hormone concentration, and a reduction in follistatin concentration. A significant reduction in heart rate was observed only in the blood flow restriction arm (−15.00 beats·min^−1^; −27.86 to −2.14; *p* = 0.027). No significant between-group differences were observed for any of these parameters.

Bone turnover markers were reported by one study. De Sá et al. [36] reported an increase in C-terminal telopeptide of type I collagen in both arms (BFR: ES 1.13; 0.43 to 1.70; HL: ES 0.59; −0.05 to 1.08), with no changes in bone-specific alkaline phosphatase or osteocalcin and no between-group differences.

None of the nine included studies reported markers of mitochondrial adaptation, capillary density, or electromyographic measures. The body of evidence included in this review therefore does not permit conclusions regarding molecular mechanisms or neuromuscular adaptations assessed directly.

### 3.6. Certainty of Evidence

The certainty of evidence was assessed using the GRADE approach [29] for five outcome groups, according to the criteria described in Section 2.8 [30]. Certainty was low for muscle mass and cross-sectional area and very low for dynamic maximal strength, isometric strength, isokinetic torque, and functional performance. The reasons for each downgrade are given in the footnotes to Table 7. Because publication bias could not be assessed formally, these ratings represent upper bounds.

### 3.7. Publication Bias and Small-Study Effects

According to the recommendations of the Cochrane Handbook [25], graphical and statistical methods for detecting small-study effects require at least ten studies within a given comparison. Nine studies were included in the present review, and effect sizes were calculated for seven of them. Therefore, funnel plot construction and tests of asymmetry were not performed.

The risk of publication bias was assessed qualitatively. Factors considered to increase this risk included:

▪ Small sample sizes across all studies;
▪ Lack of prospective registration in most studies;
▪ The presence of companion publications reporting selected endpoints from the same cohort in separate papers [41,42];
▪ No search of clinical trial registries, which precluded the identification of registered but unpublished trials.

Factors considered to reduce the risk included:

▪ Inclusion of gray literature and doctoral theses in the search strategy;
▪ No language restrictions;
▪ The presence of studies reporting statistically non-significant findings and results that were inconsistent with the authors’ expectations [37,39].

## 4. Discussion

This systematic review indicates that the relative effectiveness of blood flow restriction training and high-load resistance training in older adults may differ depending on the outcome assessed. For muscle mass and cross-sectional area, no clear difference between modalities was observed; however, given the wide confidence intervals, this finding should not be interpreted as evidence of equivalence [43]. Findings for muscle strength were less homogeneous, with point estimates more often in the direction of high-load resistance training, although most confidence intervals included no difference. This pattern therefore suggests that the evaluation of BFR as an alternative to high-load resistance training should take into account the type of adaptation being assessed. Interpretation requires caution, because the certainty of evidence was low for muscle mass and very low for all strength and functional outcomes.

### 4.1. Muscle Strength Adaptations and Test Specificity

The differences in muscle strength observed in this review are largely consistent with findings from previous meta-analyses. Lixandrão et al. demonstrated greater strength gains following high-load resistance training than following low-load BFR training, with similar effects of both modalities on muscle hypertrophy [14]. Similar observations were reported by Fabero-Garrido et al. in adults over 60 years of age, who found a small advantage of high-load resistance training for strength and no significant differences between modalities for hypertrophy [18], and by Centner et al. in healthy adults over 50 years of age [17], whereas Grønfeldt et al. found no significant difference in strength between blood flow-restricted and heavy-load training in healthy adults [16]. Most previous syntheses therefore support the discrepancy between strength and hypertrophic responses identified in the present review, although the magnitude of the strength difference appears to vary across populations and outcome measures.

When interpreting differences in strength, however, the specificity of the tests used should be considered. In six of the included studies, the primary strength outcome was assessed using a test corresponding to the exercise performed by the high-load resistance training group. The significance of this observation should be considered in the context of the task specificity of strength adaptations demonstrated for dynamic resistance training [44]. Test specificity is therefore a plausible, but untested, explanation for part of the difference observed in dynamic tests. In the present review, point estimates from dynamic strength tests were more often in the direction of high-load resistance training than those from isometric and isokinetic measures. This was particularly evident in the study by Cook et al., in which, in the same participants, the effect size was g = 1.14 for the dynamic 1RM test and g = 0.40 for the isometric assessment [37]. At the same time, the difference between the median effect sizes for dynamic tests and isometric and isokinetic tests across the entire evidence base was small (0.49 and 0.40, respectively), and this comparison was descriptive. Moreover, Lixandrão et al. found that high-load training remained superior when strength was assessed with non-specific tests [14]. The role of test specificity should therefore be regarded as hypothesis-generating and should be examined in trials using tests that are not specific to the training modality of either arm.

### 4.2. Muscle Mass and Hypertrophic Adaptations

In contrast to the findings for muscle strength, analysis of changes in muscle mass and cross-sectional area did not indicate a clear advantage of either of the compared training modalities. The calculated effect sizes were small, and their confidence intervals included no difference. This finding is consistent with previous evidence syntheses. Centner et al. found no significant difference in muscle mass between LL-BFR and high-load resistance training in older adults [17], while Fabero-Garrido et al. also found no significant difference in hypertrophy between these modalities in adults over 60 years of age [18]. In the present review, however, the confidence intervals were wide, and the absence of a statistically significant difference does not demonstrate that the two modalities produce equivalent hypertrophy [43].

The discrepancy between hypertrophic responses and changes in strength was also evident at the individual study level. In the study by Cook et al., the advantage of high-load resistance training in the dynamic strength test (g = 1.14) was not accompanied by a corresponding difference in quadriceps muscle cross-sectional area (g = −0.15) [37], whereas in the study by Vechin et al., a point estimate in the direction of high-load resistance training for strength (g = 0.43), with a confidence interval including no difference, was accompanied by virtually no difference in muscle cross-sectional area (g = 0.01) [32]. These observations suggest that differences in strength gains between the compared modalities cannot be interpreted solely on the basis of changes in muscle size. Neural adaptations, which account for part of the strength gains after resistance training, represent one possible explanation. However, none of the included studies reported electromyographic or other neurophysiological measures; neural mechanisms therefore cannot be inferred from the present data, and differences in test familiarization, measurement error, or muscle quality may contribute equally.

### 4.3. Blood Flow Restriction Dose and Methodological Heterogeneity

The dosing of blood flow restriction varied across the studies included in this review. Pressure was individualized relative to arterial occlusion pressure (AOP) in six trials, indexed to systolic blood pressure in two, and fixed in one, and the dose was reported completely in five of the nine trials. This variation is relevant to the comparability of protocols because the actual degree of restriction depends not only on the applied pressure but also on cuff characteristics, including cuff width [10]. Patterson et al. therefore recommend individualizing pressure relative to AOP, indicating a range of 40–80% AOP as being used in BFR resistance training [9]. Consequently, BFR should not be regarded as a single, uniform intervention. In particular, the finding for muscle mass was derived mainly from protocols using 50% AOP and wide cuffs [32,33,39] and cannot be generalized to other pressures, cuff widths, or methods of pressure prescription.

A distinction should also be made between methodological variability and factors supported by the data as effect modifiers. Differences in pressure prescription, session frequency, and intervention duration represent variability between protocols; however, the available data do not indicate that any of these parameters modified the difference between BFR and high-load training. The only evidence from a within-trial comparison came from Letieri et al. [40], in which the lower-pressure group showed a larger difference favoring high-load training than the higher-pressure group; this exploratory comparison was based on small samples, and all confidence intervals included no difference. In a larger meta-analysis comparing BFR with high-load training, training status moderated the difference in strength, whereas training duration and frequency did not [15]. Whether pressure magnitude modifies the relative effectiveness of BFR in older adults therefore remains to be established in adequately powered trials.

### 4.4. Healthy Aging Versus Clinical and Functionally Limited Populations

The included trials represented two distinct populations. In the four trials of healthy older adults [32,33,38,40], calculable strength estimates ranged from g = 0.32 to 0.79 in the direction of high-load training, with all confidence intervals including no difference, and no difference in quadriceps cross-sectional area was observed [32,33]. In the five trials of clinically or functionally limited populations [34,35,36,37,39], findings were more variable, ranging from a larger strength gain after BFR in women with knee osteoarthritis [35] to the largest estimate in the direction of high-load training in older adults at risk of mobility limitation [37]. Each clinical condition was represented by a single trial, which precludes conclusions for specific diagnoses. Importantly, all participants were, by design, eligible for high-load training; thus, the population for whom BFR is primarily proposed, namely individuals in whom high loads are contraindicated or poorly tolerated, was not represented. The trial in women with knee osteoarthritis [35] came closest to this scenario and reported less knee pain during BFR exercise than during high-load exercise.

### 4.5. Functional Outcomes and Clinical Implications

Evidence concerning functional performance was more limited than the data relating to strength and muscle size. Functional endpoints were reported in three studies, and the findings were heterogeneous. Cook et al. [37] found no significant change in the overall SPPB score, whereas Zhang et al. [39] reported an improvement in SPPB only in the high-load resistance training group. Bryk et al. [35], in contrast, demonstrated improvements in TUG and the Lequesne index in both groups, with no significant between-group differences. The available data therefore do not indicate a consistent direction of differences between BFR and high-load resistance training in terms of functional performance. The limited ability to draw conclusions in this area is consistent with a previous meta-analysis by Labata-Lezaun et al., which found no significant differences in physical performance between BFR and the comparator groups analyzed [19].

Given the heterogeneity of the measures used and the very low certainty of evidence, the findings of the present review do not allow a definitive determination of the relative effectiveness of BFR compared with high-load resistance training in adults over 60 years of age. For clinical practice, the direct evidence from the nine included trials should be distinguished from physiological rationale and from evidence obtained in other populations. The direct evidence shows no clear difference in muscle mass and a tendency toward larger dynamic strength gains after high-load training in older adults who were able to perform high-load training. The proposition that LL-BFR is useful for individuals in whom high loads are contraindicated or poorly tolerated rests on physiological rationale and on evidence from musculoskeletal rehabilitation and hemodynamic studies [11,12,13], rather than on trials in this population. LL-BFR may therefore be considered cautiously, under supervision and with individualized pressure, when high-load training is unsuitable, but it should not be regarded as interchangeable with high-load resistance training, particularly when strength is the primary goal. It is also important to distinguish changes in muscle mass and strength from functional performance. Previous evidence indicates that, in older adults, physical performance is more strongly associated with muscle strength than with muscle mass alone [45]. In the present review, however, the number of studies assessing functional performance was too small to determine the relationship between changes in muscle parameters and functional test outcomes. Future studies comparing LL-BFR with high-load resistance training should therefore include standardized measures of both muscle strength and functional performance.

### 4.6. Tolerability and Safety Considerations

Training tolerability is an important aspect when evaluating the potential application of BFR in older adults. The ability to induce muscular adaptations with lower external loads represents a potential advantage of LL-BFR, particularly when the use of high-load resistance training is limited or poorly tolerated. However, lower mechanical loading does not necessarily imply better tolerability of the overall procedure. In the study by Kim et al. [38], 4 of 13 participants (31%) allocated to BFR discontinued participation because of discomfort associated with the protocol, whereas in the trial by Bryk et al. [35], pain during exercise was lower with BFR than with high-load training. Adverse events were explicitly reported in only four of the nine trials [33,36,37,39], none of which described intervention-related adverse events, and no trial assessed markers of hemostasis. Safety of BFR is a separate issue and should not be assessed solely on the basis of procedural tolerability. A meta-analysis including 12 randomized studies and 378 participants found that LL-BFR was associated with acute increases in heart rate and systolic and diastolic blood pressure; however, in comparative analyses, the magnitude of these responses did not differ significantly from those observed during high-load resistance training [12]. These external data cannot substitute for systematic adverse event monitoring in older adults with chronic disease, and the data available in the present review do not allow a reliable comparison of the incidence of adverse events between LL-BFR and high-load resistance training.

### 4.7. Limitations and Future Research

This review has several limitations that should be considered when interpreting the findings. The available evidence base was limited, and the relatively small sample sizes of individual groups restricted the precision of effect estimates. The included studies also involved heterogeneous populations of older adults, including both healthy individuals and individuals with different medical conditions. This increases the clinical scope of the available evidence but, given the current number of studies, does not allow reliable determination of whether the relative effectiveness of LL-BFR and high-load resistance training differs between specific populations.

An important limitation was also the heterogeneity of training protocols and outcome assessment methods. Variation was present in training parameters and BFR dosing, as well as in the strength tests and methods used to assess muscle morphology. In addition, no equivalence or non-inferiority margin was prespecified, so the review cannot establish equivalence between modalities, and the results of two trials from the same research group [32,33] are not fully independent.

Several limitations relate to the review process. Title and abstract screening was performed by a single reviewer; single screening misses a median of approximately 5% of eligible studies [46], although the liberal inclusion rule for uncertain records and the screening of reference lists may have reduced this risk. Data were extracted by one reviewer and verified by a second, rather than extracted independently in duplicate. Clinical trial registries were not searched, which may have reduced the completeness of the search and precluded the assessment of unpublished trials. Finally, because the age criterion was applied to the sample mean, individuals younger than 60 years may have been included in four trials [32,34,35,36].

Future trials should enroll older adults in whom high-load training is contraindicated or poorly tolerated, prespecify equivalence or non-inferiority margins, individualize and fully report the BFR dose, assess strength with both specific and non-specific tests, and record adverse events systematically.

## 5. Conclusions

Evidence from nine randomized trials showed no clear difference between low-load blood flow restriction training and high-load resistance training in changes in muscle mass and cross-sectional area in older adults; because the confidence intervals were wide, this finding does not establish equivalence.

For dynamic maximal strength, the study-level point estimates available from four trials were in the direction of high-load resistance training, but confidence intervals were wide, and only one excluded no difference, and isometric and isokinetic findings were inconsistent. Strength outcomes therefore appear less consistent than those for muscle mass.

The certainty of evidence was low for muscle mass and cross-sectional area and very low for dynamic, isometric, and isokinetic strength and for functional performance; because publication bias could not be assessed, these ratings represent upper bounds.

Low-load blood flow restriction training may be considered cautiously, under supervision, as an option for older adults in whom loads of approximately 70–80% of one-repetition maximum are contraindicated or poorly tolerated. This suggestion rests mainly on physiological rationale and on evidence from other populations, because none of the included trials enrolled such individuals; the method should not be considered interchangeable with high-load resistance training, particularly for muscle strength.

Future trials should include larger samples of clinically relevant populations, prespecify equivalence margins, individualize and completely report the restriction dose, and assess strength with tests that are not specific to the training modality of either arm.

## Figures and Tables

**Table 1:** Characteristics of previous systematic reviews with meta-analyses comparing low-load blood flow restriction training with high-load resistance training.

Review	Population	Comparator(s)	Studies (Participants)	Outcomes	Main Findings for LL-BFR vs. HL	Main Difference from the Present Review
Lixandrão et al., 2018 [14]	Adults of all ages	HL (>65% 1RM)	12 (*n* = 460) for strength; 10 (*n* = 368) for hypertrophy	Strength; muscle mass	Strength favored HL (ES 0.63; 0.43 to 0.80); no significant difference in hypertrophy (ES 0.10; −0.10 to 0.30)	Not restricted to older adults
Centner et al., 2019 [17]	Healthy adults > 50 years	HL; LL-RT; walking without BFR	11 (*n* = 238)	Strength; hypertrophy	vs. HL: strength favored HL (SMD −0.42; −0.70 to −0.14); no significant difference in hypertrophy (SMD 0.21; −0.14 to 0.56)	Healthy participants only; mixed comparators; resistance and walking BFR combined
Grønfeldt et al., 2020 [16]	Healthy, habitually active adults aged 20–80 years	Heavy-load RT	16 (*n* = 310)	Strength	No significant difference in strength (SMD −0.17; −0.40 to 0.05)	Not restricted to older adults; strength only
Labata-Lezaun et al., 2022 [19]	Healthy adults ≥ 60 years	Conventional RT; high-intensity RT; no training	10 RCTs (*n* = 278)	Strength; physical performance	No significant difference in strength vs. high-intensity RT; no significant difference in physical performance	Healthy participants only; no muscle mass outcome; mixed comparators
Fabero-Garrido et al., 2022 [18]	Healthy adults > 60 years	HL (≥70% 1RM); LL-RT	14 (*n* = 340); 8 vs. HL	Strength; hypertrophy	vs. HL: strength favored HL (SMD −0.23; −0.41 to −0.05); no significant difference in hypertrophy (SMD 0.08; −0.22 to 0.38)	Healthy participants only; body circumference accepted as a hypertrophy outcome
Geng et al., 2024 [15]	Younger and older adults	HL (>65% 1RM)	51 (*n* = 1164) for strength; 28 (*n* = 703) for hypertrophy	Strength; hypertrophy; moderators	Strength favored HL (SMD −0.34; −0.52 to −0.16); no significant difference in hypertrophy (SMD −0.07; −0.21 to 0.07)	Not restricted to older adults; age examined only as a moderator

LL-BFR—low-load resistance training with blood flow restriction; HL—high-load resistance training; LL-RT—low-load resistance training without blood flow restriction; RT—resistance training; 1RM—one-repetition maximum; ES—effect size; SMD—standardized mean difference; RCT—randomized controlled trial. Effect sizes (95% confidence intervals) are reproduced with the sign convention of the original reviews.

**Table 2:** Eligibility criteria according to the PICOS framework.

	Inclusion Criteria
Population	Mean age of the studied sample ≥60 years, community-dwelling; healthy individuals, with sarcopenia as well as individuals with stable chronic diseases (criterion applied to the sample mean; individual participants younger than 60 years were not excluded)
Intervention	Blood flow restriction (BFR) training lasting ≥4 weeks, performed at least twice a week, using external pneumatic or elastic compression proximally on the limb
Comparator	High-load resistance training (≥60% 1RM) The presence of additional arms was not an exclusion criterion; however, these arms were not included in the main comparison.
Outcomes	Muscle strength as well as muscle mass and cross-sectional area
Study design	Randomized controlled trials and non-randomized controlled trials (both designs eligible a priori; no non-randomized trial met the remaining criteria)
	Exclusion criteria
1.	Interventions lasting <4 weeks or performed less frequently than 2 times a week and studies assessing solely the acute effect of a single session
2.	Lack of a parallel high-load resistance training arm
3.	Ischemic pre- and postconditioning
4.	Mean age of the studied sample <60 years
5.	Single-arm studies, case reports and case series, protocols without results, conference abstracts without available full-text data, and narrative and systematic reviews.

**Table 4:** Characteristics of the blood flow restriction dose.

Study	Cuff Width (cm)	Pressure (mmHg)	AOP (%)	Method Used to Determine Pressure	Pressure Maintained During Rest Intervals	Reporting Completeness
Vechin et al. [32]	18	71	50	Doppler	Yes	Complete
Libardi et al. [33]	17.5	67 ± 8	50	Doppler	Yes	Complete
Silva et al. [34]	18	104.2 ± 7.9	80	Doppler	No	Complete
Bryk et al. [35]	NR	200	NR	Arbitrary value	Yes	Incomplete
Kim et al. [38]	16 × 30	160	NR	130% of systolic blood pressure	Yes	Complete
Cook et al. [37]	6 × 83	184 ± 25	66	1.5 × systolic blood pressure	Yes	Complete
Letieri et al. [40]	NR	185.8 ± 5.5 (high)/105.5 ± 6.5 (low)	80	Doppler	Yes	Incomplete
Zhang et al. [39]	14	NR	50	Doppler	Yes	Incomplete
De Sá et al. [36]	7.5	NR	70	Doppler	No	Incomplete

AOP—arterial occlusion pressure; Doppler—pressure determined individually using the Doppler method; NR—not reported.

**Table 5:** Muscle strength outcomes.

Study	Test (Unit)	BFR Pre	BFR Post	HL Pre	HL Post	Change BFR	Change HL	Hedges’ g (95% CI)
Vechin et al. [32]	Leg press 1RM (kg)	273 ± 114	316 ± 141	177 ± 104	266 ± 140	+15.8%	+50.3%	0.43 (−0.56 to 1.42)
Zhang et al. [39]	Estimated knee extension 1RM (kg)	47.30 ± 6.17	51.48 ± 5.28	42.02 ± 8.41	46.41 ± 5.37	+8.8%	+10.4%	0.04 (−0.82 to 0.90)
Cook et al. [37]	Knee extension 1RM (kg)	NR	NR	NR	NR	+9.1 (5.0 to 13.2)	+21.2 (13.0 to 29.5)	1.14 (0.27 to 2.01)
Cook et al. [37]	Leg press 1RM (kg)	NR	NR	NR	NR	+18.7 (9.0 to 28.4)	+31.7 (13.6 to 50.0)	0.55 (−0.27 to 1.36)
Cook et al. [37]	Knee flexor 1RM (kg)	NR	NR	NR	NR	+5.4 (0.5 to 10.2)	+8.2 (5.4 to 11.1)	0.43 (−0.38 to 1.24)
Silva et al. [34]	Knee extension 1RM (kg)	35.85 ± 6.72	40.10 ± 7.39	27.78 ± 3.45	37.37 ± 4.58	+11.8%	+34.5%	1.07 (−0.28 to 2.41)
Libardi et al. [33]	Leg press 1RM (kg)	NR	NR	NR	NR	+35.4%	+38.1%	NR
De Sá et al. [36]	Leg press 1RM (kgf)	NR	NR	NR	NR	+33.5% (ES 1.70; 0.85 to 2.34)	+29.7% (ES 2.18; 1.10 to 2.93)	NR
Cook et al. [37]	Extensor MVIC (N·m)	NR	NR	NR	NR	+11.2 (−2.7 to 25.0)	+19.3 (8.3 to 30.3)	0.40 (−0.41 to 1.21)
Letieri et al. [40]	Isokinetic extensor torque, right (N·m)	94.14 ± 15.87	114.59 ± 14.83	91.70 ± 13.48	116.01 ± 14.50	+21.7%	+26.5%	0.32 (−0.43 to 1.08)
Letieri et al. [40]	Isokinetic extensor torque, left (N·m)	93.50 ± 15.86	114.17 ± 15.52	89.40 ± 13.04	116.57 ± 13.70	+22.1%	+30.4%	0.55 (−0.21 to 1.31)
Bryk et al. [35]	Quadriceps MVC (kg/body mass × 100)	23.2 ± 8.4	40.0 ± 9.2	24.1 ± 10.1	33.5 ± 12.9	+16.8 ± 10.3	+9.4 ± 8.3	−0.77 (−1.47 to −0.07)
Kim et al. [38]	Handgrip MVC (kg)	33.4 ± 4.7	36.3 ± 4.7	34.0 ± 4.4	39.8 ± 4.4	+8.7%	+17.1%	0.79 (−0.15 to 1.73)

BFR—blood flow restriction arm; HL—high-load resistance training arm; values are presented as mean ± standard deviation; values in parentheses for changes indicate the 95% confidence interval reported by the authors; ES—within-group effect size reported by the authors; NR—not reported in a form allowing entry into the table; Hedges’ g—standardized mean difference in change from baseline, with positive values indicating an advantage of high-load resistance training.

**Table 6:** Muscle mass and cross-sectional area outcomes.

Study	Measurement Method (Unit)	BFR Pre	BFR Post	HL Pre	HL Post	Change BFR	Change HL	Hedges’ g (95% CI)
Vechin et al. [32]	Quadriceps cross-sectional area, MRI (cm^2^)	67.4 ± 21.5	71.4 ± 22.1	56.9 ± 14.9	61.1 ± 14.8	+6.6%	+7.9%	0.01 (−0.97 to 0.99)
Zhang et al. [39]	Appendicular skeletal muscle mass index, BIA (kg·m^−2^)	6.16 ± 0.73	6.28 ± 0.81	6.33 ± 0.84	6.63 ± 0.97	+0.13 (−0.10 to 0.36)	+0.30 (0.00 to 0.59)	0.42 (−0.45 to 1.29)
Cook et al. [37]	Quadriceps cross-sectional area, MRI (cm^2^)	NR	NR	NR	NR	+3.23 (1.29 to 5.16)	+2.86 (1.87 to 3.86)	−0.15 (−0.95 to 0.65)
Libardi et al. [33]	Quadriceps cross-sectional area, MRI (cm^2^)	NR	NR	NR	NR	+7.6%	+7.3%	NR
Kim et al. [38]	Forearm circumference (cm)	26.3 ± 1.1	26.7 ± 1.1	25.9 ± 1.0	26.1 ± 1.0	+1.5%	+0.8%	Outcome did not qualify

BFR—blood flow restriction arm; HL—high-load resistance training arm; MRI—magnetic resonance imaging; BIA—bioelectrical impedance analysis; NR—not reported. Values are presented as mean ± standard deviation; values in parentheses for changes indicate the 95% confidence interval reported by the authors. Percentage changes are presented as reported by the authors; for Vechin et al. [32].

**Table 7:** Certainty of evidence assessment.

Outcome	Number of Studies (Participants)	Risk of Bias	Inconsistency	Indirectness	Imprecision	Publication Bias	Range of Study-Level Effect Sizes, g	Certainty
Maximal strength (1RM)	6 studies (*n* = 113)	Serious ^a^	Serious ^b^	Not serious	Serious ^c^	Undetermined ^d^	g from 0.04 to 1.14 (four trials)	●○○○ VERY LOW
Isometric strength (MVC/MVIC)	3 studies (*n* = 77)	Serious ^a^	Serious ^e^	Serious ^f^	Serious ^c^	Undetermined ^d^	g from −0.77 to 0.79	●○○○ VERY LOW
Isokinetic knee extensor torque	1 study (*n* = 32)	Serious ^a^	Not assessable ^g^	Not serious	Very serious ^h^	Undetermined ^d^	g = 0.32 and 0.55	●○○○ VERY LOW
Muscle mass and cross-sectional area	4 studies (*n* = 79)	Serious ^a^	Not serious ^i^	Not serious ^j^	Serious ^c^	Undetermined ^d^	g from −0.15 to 0.42 (three trials)	●●○○ LOW
Functional performance	3 studies (*n* = 79)	Serious ^a^	Serious ^k^	Not serious	Serious ^c^	Undetermined ^d^	Not calculated; heterogeneous outcomes	●○○○ VERY LOW

g—effect size expressed as Hedges’ g for the between-group comparison; positive values indicate an advantage of high-load resistance training; MVC—maximal voluntary contraction; MVIC—maximal voluntary isometric contraction. Certainty: ●●○○ low, ●○○○ very low. ^a^ Downgraded by one level for risk of bias: all contributing studies were judged as raising some concerns or at high risk of bias. ^b^ Downgraded for inconsistency: all point estimates were in the direction of high-load training, but their magnitudes ranged from trivial (g = 0.04) to large (g = 1.14) without a plausible explanation, based on only four trials with calculable estimates. ^c^ Downgraded for imprecision: the total sample was below approximately 400 participants (pragmatic criterion; Section 2.8), and most confidence intervals included both no difference and effects of |g| ≥ 0.5. ^d^ Not formally assessable because fewer than ten studies contributed to each outcome; not used for downgrading, and the ratings should be regarded as upper bounds. ^e^ Downgraded for inconsistency: point estimates differed in direction, and the confidence interval of one trial excluded no difference in favor of BFR (g = −0.77; −1.47 to −0.07) [35]. ^f^ Downgraded for indirectness: one trial assessed handgrip strength after training of the forearm muscles only [38], in contrast to the lower-limb training and outcomes of the remaining trials. ^g^ Not assessable: evidence from a single trial. ^h^ Downgraded by two levels for imprecision: a single trial (*n* = 32) with confidence intervals including no difference. ^i^ Not downgraded: estimates were trivial to small and consistently compatible with no difference. ^j^ Not downgraded: three of four trials assessed quadriceps cross-sectional area by magnetic resonance imaging; one trial used the appendicular skeletal muscle mass index estimated by bioelectrical impedance [39]. ^k^ Downgraded for inconsistency: heterogeneous instruments with an inconsistent pattern of within- and between-group findings.

## Data Availability

The original contributions presented in the study are included in the article; further inquiries can be directed to the corresponding author.

## Supplementary Materials

The following supporting information can be downloaded at: https://www.mdpi.com/article/10.3390/jcm15208079/s1, Table S1: PRISMA 2020 checklist.

## Appendix A

EBSCOhost:
TI (elder* OR older OR aged OR aging OR ageing OR senior* OR geriatric* OR sarcopeni* OR frail* OR “old age” OR “oldest old” OR septuagenarian* OR octogenarian* OR nonagenarian* OR centenarian* OR postmenopaus* OR “late life” OR “community dwelling” OR “nursing home*” OR “long term care”) OR AB (elder* OR older OR aged OR aging OR ageing OR senior* OR geriatric* OR sarcopeni* OR frail* OR “old age” OR “oldest old” OR septuagenarian* OR octogenarian* OR nonagenarian* OR centenarian* OR postmenopaus* OR “late life” OR “community dwelling” OR “long term care”)
TI ((blood N2 flow N2 restrict*) OR (blood N2 flow N2 occlu*) OR “blood flow restriction” OR “restricted blood flow” OR “flow restriction” OR BFR OR BFRT OR BFRE OR KAATSU OR “vascular occlusion” OR “vascular restriction” OR “venous occlusion” OR “partial occlusion” OR (occlusi* N2 (training OR exercise)) OR (isch#emic N2 (training OR exercise)) OR “cuff occlusion” OR “pressure cuff*” OR “limb occlusion pressure” OR “arterial occlusion pressure” OR tourniquet*) OR AB ((blood N2 flow N2 restrict*) OR (blood N2 flow N2 occlu*) OR “blood flow restriction” OR “restricted blood flow” OR “flow restriction” OR BFR OR BFRT OR BFRE OR KAATSU OR “vascular occlusion” OR “vascular restriction” OR “venous occlusion” OR “partial occlusion” OR (occlusi* N2 (training OR exercise)) OR (isch#emic N2 (training OR exercise)) OR “cuff occlusion” OR “pressure cuff*” OR “limb occlusion pressure” OR “arterial occlusion pressure” OR tourniquet*)
TI (exercis* OR train* OR rehabilitat* OR physiotherap* OR “physical therapy” OR “physical activity” OR “low load” OR “low intensity” OR walking OR “muscle strength” OR hypertroph*) OR AB (exercis* OR train* OR rehabilitat* OR physiotherap* OR “physical therapy” OR “physical activity” OR “low load” OR “low intensity” OR walking OR “muscle strength” OR hypertroph*)
Web of Science:
TS = (elder* OR older OR aged OR aging OR ageing OR senior* OR geriatric* OR sarcopeni* OR presarcopeni* OR frail* OR “old age” OR “oldest old” OR “advanced age” OR septuagenarian* OR octogenarian* OR nonagenarian* OR centenarian* OR postmenopaus* OR “late life” OR “community dwelling” OR “nursing home*” OR “long term care” OR “assisted living”)
TS = (“blood flow restrict*” OR “blood-flow restrict*” OR “restricted blood flow” OR “restriction of blood flow” OR “blood flow occlusion” OR “flow restrict*” OR (blood NEAR/2 flow NEAR/2 (restrict* OR occlu*)) OR BFR OR BFRT OR BFRE OR “LL-BFR” OR KAATSU OR “kaatsu train*” OR “vascular occlusion” OR “vascular restrict*” OR “venous occlusion” OR “partial occlusion” OR “partial vascular occlusion” OR “occlusion train*” OR “occlusive train*” OR “occlusion exercise” OR “ischemic train*” OR “ischaemic train*” OR “ischemic exercise” OR “ischaemic exercise” OR “cuff occlusion” OR “pressure cuff*” OR “limb occlusion pressure” OR “arterial occlusion pressure” OR tourniquet*)
TS = (exercis* OR train* OR “resistance train*” OR “strength train*” OR “weight train*” OR rehabilitat* OR physiotherap* OR “physical therapy” OR “physical activity” OR “low load” OR “low-load” OR “low intensity” OR “low-intensity” OR walking OR “muscle strength” OR hypertroph*)
PubMed:
“Aged”[Mesh] OR “Aging”[Mesh] OR “Geriatrics”[Mesh] OR “Health Services for the Aged”[Mesh] OR “Sarcopenia”[Mesh] OR “Frailty”[Mesh] OR “Frail Elderly”[Mesh] OR “Independent Living”[Mesh] OR “Homes for the Aged”[Mesh] OR “Nursing Homes”[Mesh] OR elder*[tiab] OR older[tiab] OR aged[tiab] OR aging[tiab] OR ageing[tiab] OR senior*[tiab] OR geriatric*[tiab] OR septuagenarian*[tiab] OR octogenarian*[tiab] OR nonagenarian*[tiab] OR centenarian*[tiab] OR “old age”[tiab] OR “oldest old”[tiab] OR “advanced age”[tiab] OR sarcopeni*[tiab] OR presarcopeni*[tiab] OR frail*[tiab] OR “late life”[tiab] OR postmenopaus*[tiab] OR “community dwelling”[tiab] OR “nursing home”[tiab] OR “nursing homes”[tiab] OR “long term care”[tiab] OR “assisted living”[tiab]
“blood flow restriction”[tiab] OR “blood flow restricted”[tiab] OR “blood flow restrictive”[tiab] OR “blood-flow restriction”[tiab] OR “restricted blood flow”[tiab] OR “restriction of blood flow”[tiab] OR “blood flow occlusion”[tiab] OR “occlusion of blood flow”[tiab] OR “flow restriction”[tiab] OR “flow restricted”[tiab] OR BFR[tiab] OR BFRT[tiab] OR BFRE[tiab] OR “LL-BFR”[tiab] OR “low load blood flow”[tiab] OR KAATSU[tiab] OR “kaatsu training”[tiab] OR “vascular occlusion”[tiab] OR “vascular restriction”[tiab] OR “venous occlusion”[tiab] OR “partial occlusion”[tiab] OR “partial vascular occlusion”[tiab] OR “occlusion training”[tiab] OR “occlusive training”[tiab] OR “occlusion exercise”[tiab] OR “ischemic training”[tiab] OR “ischaemic training”[tiab] OR “ischemic exercise”[tiab] OR “ischaemic exercise”[tiab] OR “cuff occlusion”[tiab] OR “pressure cuff”[tiab] OR “pressure cuffs”[tiab] OR “limb occlusion pressure”[tiab] OR “arterial occlusion pressure”[tiab] OR “Tourniquets”[Mesh] OR tourniquet*[tiab]
“Resistance Training”[Mesh] OR “Exercise”[Mesh] OR “Exercise Therapy”[Mesh] OR “Physical Conditioning, Human”[Mesh] OR “Exercise Movement Techniques”[Mesh] OR “Muscle Strength”[Mesh] OR “Walking”[Mesh] OR “Rehabilitation”[Mesh] OR exercis*[tiab] OR train*[tiab] OR rehabilitat*[tiab] OR physiotherap*[tiab] OR “physical therapy”[tiab] OR “physical activity”[tiab] OR “low load”[tiab] OR “low-load”[tiab] OR “low intensity”[tiab] OR “low-intensity”[tiab] OR walking[tiab] OR “muscle strength”[tiab] OR hypertroph*[tiab] OR “strength gains”[tiab]
Scopus:
TITLE-ABS-KEY (elder* OR older OR aged OR aging OR ageing OR senior* OR geriatric* OR sarcopeni* OR frail* OR “old age” OR “oldest old” OR “advanced age” OR septuagenarian* OR octogenarian* OR nonagenarian* OR centenarian* OR postmenopaus* OR “late life” OR “community dwelling” OR “nursing home*” OR “long term care” OR “assisted living”) AND TITLE-ABS-KEY ((blood W/2 flow W/2 restrict*) OR (blood W/2 flow W/2 occlu*) OR “blood flow restriction” OR “restricted blood flow” OR “restriction of blood flow” OR “flow restriction” OR BFR OR BFRT OR BFRE OR KAATSU OR “vascular occlusion” OR “vascular restriction” OR “venous occlusion” OR “partial occlusion” OR (occlusi* W/2 (training OR exercise)) OR (ischemic W/2 (training OR exercise)) OR (ischaemic W/2 (training OR exercise)) OR “cuff occlusion” OR “pressure cuff*” OR “limb occlusion pressure” OR “arterial occlusion pressure” OR tourniquet*) AND TITLE-ABS-KEY (exercis* OR train* OR rehabilitat* OR physiotherap* OR “physical therapy” OR “physical activity” OR “low load” OR “low intensity” OR walking OR “muscle strength” OR hypertroph*)
